# Low-dose aspirin was associated with an increased risk of cardiovascular events in patients with chronic kidney disease and low bodyweight: results from KNOW-CKD study

**DOI:** 10.1038/s41598-021-86192-4

**Published:** 2021-03-23

**Authors:** Yun Jung Oh, Ae Jin Kim, Han Ro, Jae Hyun Chang, Hyun Hee Lee, Wookyung Chung, Young Youl Hyun, Joongyub Lee, Yeong Hoon Kim, Seung Hyeok Han, Dong-Wan Chae, Curie Ahn, Kook-Hwan Oh, Ji Yong Jung

**Affiliations:** 1 Department of Internal Medicine, Graduate School of Medicine, Gachon University, Incheon, Republic of Korea; 2 Division of Nephrology, Department of Internal Medicine, Cheju Halla General Hospital, Cheju, Republic of Korea; 3 Division of Nephrology, Department of Internal Medicine, Gachon University Gil Medical Center, Gachon University College of Medicine, 21, Namdong-daero 774 beon-gil, Namdong-gu, Incheon, 21565 Republic of Korea; 4 College of Medicine, Gachon University, Incheon, Republic of Korea; 5 Department of Internal Medicine, Sungkyunkwan University School of Medicine, Kangbuk Samsung Hospital, Seoul, Republic of Korea; 6 Department of Prevention and Management, School of Medicine, Inha University, Incheon, Republic of Korea; 7 Department of Internal Medicine, College of Medicine, Busan Paik Hospital, Inje University, Busan, Republic of Korea; 8 Department of Internal Medicine, College of Medicine, Institute of Kidney Disease Research, Yonsei University, Seoul, Republic of Korea; 9 Department of Internal Medicine, Seoul National University Bundang Hospital, Seoul, Republic of Korea; 10 Department of Internal Medicine, Seoul National University Hospital, Seoul, Republic of Korea

**Keywords:** Nephrology, Kidney diseases

## Abstract

The benefits and risks of aspirin therapy for patients with chronic kidney disease (CKD) who have a high burden of cardiovascular events (CVE) are controversial. To examine the effects of low-dose aspirin on major clinical outcomes in patients with CKD. As a prospective observational cohort study, using propensity score matching, 531 aspirin recipients and non-recipients were paired for analysis from 2070 patients and fulfilled the inclusion criteria among 2238 patients with CKD. The primary outcome was the first occurrence of major CVE. The secondary outcomes were kidney events defined as a > 50% reduction of estimated glomerular filtration rate from baseline, doubling of serum creatinine, or onset of kidney failure with replacement therapy, the all-cause mortality, and bleeding event. The incidence of CVE was significantly greater in low-dose aspirin users than in non-users (HR 1.798; *P* = 0.011). A significant association between aspirin use and an increased risk of CVE was observed only in the lowest quartile of body weight (HR 4.014; *P* = 0.019) (Q1 < 60.0 kg). Secondary outcomes were not significantly different between aspirin users and non-users. It needs to be individualized of prescribing low-dose aspirin for the prevention of cardiovascular events in patients with chronic kidney disease, particularly patients with low bodyweight (< 60 kg).

## Introduction

Chronic kidney disease (CKD) is an important pandemic health problem with an increasing prevalence and high economic burden^1–3^. Individuals with CKD have a substantially increased risk of cardiovascular disease (CVD), compared with the general population^4,5^. CVD is known to be a leading cause of death in patients with CKD^1,6^. Therefore, the efforts to reduce CVD are essential in patients with CKD. Aspirin has been widely used to reduce cardiovascular morbidities and mortality in patients who are at high risk and have previous experiences of cardiovascular events (CVE) such as myocardial infarction or stroke. The beneficial effects of low-dose aspirin treatment for secondary prevention of CVE in people who already have cardiovascular disease (CVD) have been definitely shown in numerous studies^7–9^, but the effects of aspirin for primary prevention is less clear and remains controversial^9^. Current evidence is limited regarding the use of aspirin for primary and secondary prevention of CVD in patients with CKD. This is attributed to the systematic exclusion of patients with CKD from most previous randomized clinical trials. Additionally, the different etiological pathophysiology of CVD in patients with CKD is associated with uncertainty about the beneficial effects of aspirin treatment come from the general population.

Nowadays, the preventive effect of low-dose aspirin on CVD has been called into question with the studies showing inconsistent efficacy of the drug. Actually, according to the studies performed in diabetic patients with nephropathy or without who are at increased CVD risk, while some studies showed a beneficial effect of low-dose aspirin in reducing CVE^10,11^, the other studies showed that the use of low-dose aspirin was not associated with the reduction of CVE risk^12–14^. Moreover, a recent study reported that the effect of aspirin on the prevention of CVE was different depending on the body size, so claimed that a one-dose-fits-all approach should be avoided^15^. The study showed that effectiveness of low dose aspirin disappeared at larger body size and the effect modification by weight was remained in men and women, elderly, and people with diabetes. Given that kidney function has been known to influence on bioavailability of drugs^16^ and CKD is one of the important risk factors for CVE, it is worthwhile to investigate whether the relationship between body size and efficacy of low-dose aspirin is consistent within individuals with CKD. Therefore, in this study, we sought to evaluate the effect of aspirin treatment on CVE and other clinical outcomes in the CKD population prior to dialysis, particularly the effect of low dose aspirin on body size.

## Results

### Study population and baseline characteristics

A total of 2070 patients with CKD were included in this study and the mean eGFR of study population was 53.1 ± 30.9 mL/min/1.73 m^2^. Of whom 571 (27.6%) patients were prescribed aspirin at enrollment. Almost all aspirin users (98%) were taking a low-dose (75–100 mg) of one. The other antiplatelet agents such as clopidogrel and ticlopidine were prescribed for 4.4% (92 of 2070), and warfarin was prescribed for 1.3% (27 of 2070). Baseline demographic characteristics, laboratory data, and medical history of the study population are shown in Table 1. There was a considerable imbalance of baseline characteristics between aspirin users and non-users. Using PS matching, a total of 531 aspirin users were successfully matched to non-users. After PS matching, demographic characteristics, the prevalence of medical comorbidities, and medication status were not significantly different between the two groups.

**Table 1:** Baseline characteristics of study participants.

Variable	Before matching				After propensity matching			
	Aspirin users, (N = 571)	Non-users, (N = 1499)	*P*	Standardized differences	Aspirin users, (N = 531)	Non-users, (N = 531)	*P*	Standardized differences
Age, year	58.7 ± 10.0	51.7 ± 12.5	< 0.001	0.616	58.1 ± 10.0	58.4 ± 10.3	0.609	0.030
Male gender, n (%)	401 (70.2)	864 (57.6)	< 0.001	0.264	364 (68.5)	357 (67.2)	0.692	0.028
BMI, kg/m^2^	25.1 ± 3.2	24.4 ± 3.4	< 0.001	0.237	25.1 ± 3.3	25.1 ± 3.3	0.706	0.023
Smoking, n (%)	318 (55.7)	652 (43.5)	< 0.001	0.246	282 (53.1)	278 (52.4)	0.854	0.015
Diabetes, n (%)	288 (50.4)	415 (27.7)	< 0.001	0.480	258 (48.6)	246 (46.3)	0.514	0.045
Hypertension, n (%)	564 (98.8)	1428 (95.3)	0.001	0.208	524 (98.7)	527 (99.2)	0.505	0.056
Previous CVD, n (%)	98 (17.2)	110 (7.3)	< 0.001	0.303	77 (14.5)	73 (13.7)	0.796	0.022
eGFR, mL/min/1.73 m^2^	44.9 ± 23.9	56.2 ± 32.6	< 0.001	0.396	45.6 ± 24.2	45.2 ± 26.5	0.810	0.015
UPCR, g/g	1.6 ± 2.3	1.2 ± 2.1	< 0.001	0.197	1.6 ± 2.3	1.7 ± 2.6	0.724	0.021
**Laboratory**								
Hemoglobin, g/dL	12.8 ± 2.1	12.8 ± 2.0	0.989	0.001	12.8 ± 2.1	12.8 ± 2.1	0.925	0.006
Albumin, g/dL	4.1 ± 0.4	4.2 ± 0.4	0.001	0.171	4.1 ± 0.5	4.1 ± 0.5	0.687	0.024
Cholesterol, mg/dL	168.1 ± 39.6	176.7 ± 38.8	< 0.001	0.220	169.3 ± 40.0	168.8 ± 37.6	0.856	0.011
**Medications**								
RAAS blockers, n (%)	498 (87.2)	1272 (84.9)	0.196	0.068	466 (87.8)	460 (86.6)	0.643	0.034
CCB, n (%)	304 (53.2)	588 (39.2)	< 0.001	0.284	280 (52.7)	266 (50.1)	0.405	0.053
Beta-blockers, n (%)	220 (38.5)	314 (20.9)	< 0.001	0.392	187 (35.2)	171 (32.2)	0.339	0.064
Statin, n (%)	388 (68.0)	680 (45.4)	< 0.001	0.468	350 (65.9)	351 (66.1)	1.000	0.004

Conversion factors for units were as follows: hemoglobin in g/dL to g/L, × 10; albumin in mg/dL to g/L, ×10; cholesterol in mg/dL to mmol/L × 0.02586.

*BMI* body mass index, *CVD* cardiovascular disease, *eGFR* estimated glomerular filtration rate, *UPCR* urine protein to creatinine ratio, *RAAS* renin–angiotensin–aldosterone system, *CCB* calcium channel blocker.

### Effects of aspirin on the risk of CVE in patients with CKD

During the median follow up time of 51.8 months, CVE occurred in 58 (10.2%) aspirin users versus 69 (4.6%) non-users in the unmatched cohort and 52 (9.8%) aspirin users versus 32 (6.0%) non-users in the matched cohort. Cox proportional regression analysis showed that the risk of CVE was significantly higher in aspirin users than non-users in both unmatched and matched cohort (HR 1.646; 95% CI 1.126–2.407, *P* = 0.010 in the unmatched cohort, and HR 1.798; 95% CI 1.146–2.819, *P* = 0.011 in the matched cohort) (Table 2). In order to evaluate the effects of aspirin use on primary and secondary prevention of CVE, analyses were performed by dividing the patients without a history of previous CVD and those with history, separately. The incidence of CVE was not significantly different between aspirin users and non-users in patients without a history of previous CVD (HR 1.404; 95% CI 0.900–2.192; *P* = 0.135 in the unmatched cohort, and HR 1.437; 95% CI 0.859–2.404; *P* = 0.167 in the matched cohort). However, the incidence of CVE was significantly greater in aspirin users than non-users in patients who had previously experienced CVD (HR 2.625; 95% CI 1.169–5.891; *P* = 0.019 in the unmatched cohort, and HR 3.947; 95% CI 1.343–11.598; *P* = 0.013 in the matched cohort). When analyses were conducted in subgroups stratified by eGFR ≥ 60 mL/min/1.73 m^2^ and eGFR < 60 mL/min/1.73 m^2^, the risk of CVE was significantly greater in aspirin users than non-users in patients with eGFR < 60 mL/min/1.73 m^2^ (HR 1.748; 95% CI 1.127–2.713; *P* = 0.013 in the unmatched cohort, and HR 2.143; 95% CI 1.258–3.650; *P* = 0.005 in the matched cohort) but not different between them in patients with eGFR ≥ 60 mL/min/1.73 m^2^ (Supplementary Table S1).

**Table 2:** Multivariate Cox proportional analyses for clinical outcomes^a^.

	Unmatched cohort		Matched cohort	
	HR (95% CI)	*P*	HR (95% CI)	*P*
**CVE**				
Unadjusted model	2.282 (1.610–3.236)	< 0.001	1.672 (1.076–2.597)	0.022
Adjusted model	1.646 (1.126–2.407)	0.010	1.798 (1.146–2.819)	0.011
**Renal event**				
Unadjusted model	1.281 (1.072–1.532)	0.006	1.048 (0.844–1/301)	0.674
Adjusted model	1.132 (0.934–1.372)	0.208	1.085 (0.872–1.352)	0.464
**All-cause mortality**				
Unadjusted model	1.382 (0.875–2.182)	0.165	0.648 (0.384–1.092)	0.103
Adjusted model	0.751 (0.456–1.236)	0.259	0.624 (0.360–1.079)	0.092
**Composite outcome**				
Unadjusted model	1.473 (1.257–1.726)	< 0.001	1.118 (0.921–1.358)	0.259
Adjusted model	1.174 (0.990–1.394)	0.066	1.167 (0.958–1.420)	0.125
**Bleeding events**				
Unadjusted model	1.316 (0.616–2.811)	0.479	1.349 (0.513–3.545)	0.544
Adjusted model	1.276 (0.558–2.918)	0.564	1.390 (0.517–3.741)	0.514

Adjusted for age, male gender, BMI, smoking, baseline eGFR, previous CVD, diabetes, hypertension, proteinuria, hemoglobin, albumin and total cholesterol levels, and use of medications (RAAS blockers, CCB, beta-blockers, statin, warfarin, and other antiplatelet agents.

*HR* hazards ratio, *CI* confidence interval.

### Effects of aspirin on secondary outcomes in patients with CKD

During the follow-up time, kidney event developed in 177 (31.0%) aspirin users and 381 (25.4%) non-users in the unmatched cohort and 167 (31.5%) aspirin users and 161 (30.3%) non-users in the matched cohort. Multivariable Cox regression analysis showed that use of aspirin was not associated with a risk of kidney events in both unmatched and matched cohort (Table 2). All-cause mortality was observed in 28 (4.9%) aspirin users and 54 (3.6%) non-users in the unmatched cohort and 24 (4.5%) aspirin users and 34 (6.4%) non-users in the matched cohort. All-cause mortality was not significantly different between aspirin users and non-users in both unmatched and matched cohort (Table 2). Bleeding events were observed in 10 (1.8%) aspirin users and 20 (1.3%) non-users in the unmatched cohort and 10 (1.9%) aspirin users and 7 (1.3%) non-users in the matched cohort. Bleeding risk was not significantly different between aspirin users and non-users in both unmatched and matched cohort (Table 2).

### Effects of aspirin on clinical outcomes according to bodyweight in patients with CKD

**Figure 1:**
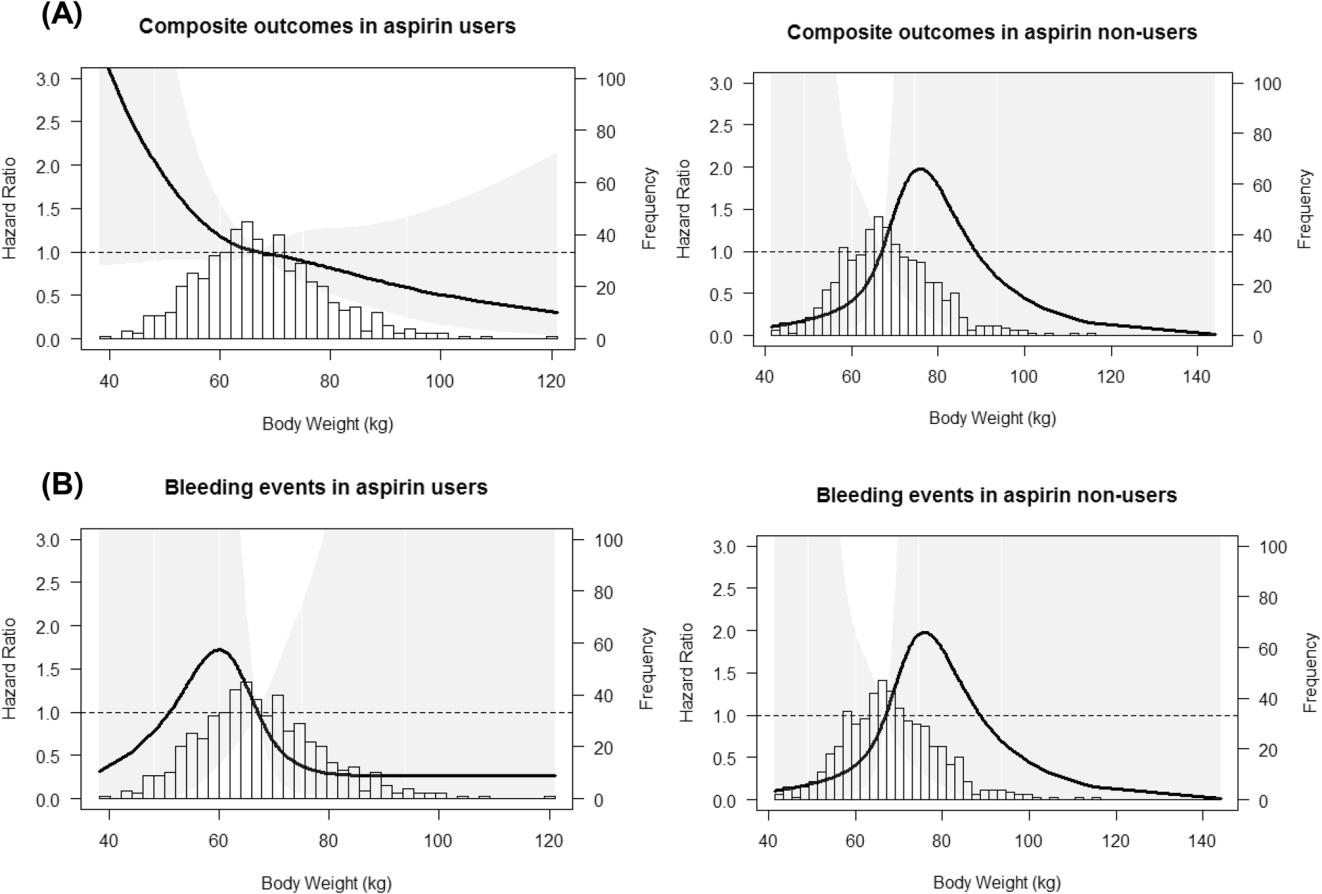
Adjusted risk of clinical outcomes according to body weight in aspirin users versus non-users after PS matching. The spine curves show the adjusted hazard ratio of (**A**) the composite outcome of the CVE or kidney event or death, and bleeding event, (**B**) bleeding events. Hazard ratios were adjusted for use of age, sex, BMI, smoking history, diabetes, hypertension, CVD, eGFRcr, uPCR, serum levels of hemoglobin, albumin, total cholesterol, and the use of RAAS blockers, CCBs, beta-blockers, statins, warfarin, and other antiplatelet agents. The histograms represent the frequency of distribution of body weight.

To investigate whether the effect of aspirin is different depending on body size, we evaluated the effect of aspirin on study outcomes by conducting multivariable Cox regression analysis stratified by the bodyweight quartiles. Analysis with both unmatched and matched cohort showed that in the lowest quartile of bodyweight, the use of aspirin was significantly associated with an increased incidence of CVE, but in the other quartiles bodyweight the use of aspirin was not associated with the risk of CVE (Table 3). The incidence of secondary outcomes was not significantly different between aspirin users and non-users across all quartiles of bodyweight in both unmatched and matched cohort, except that the risk of the composite outcome of CVE or kidney events or death was significantly increased in aspirin users who belong to the lowest quartile of bodyweight (Q1) in the matched cohort (Table 3). To determine the effects of aspirin on study outcomes according to bodyweight, we plotted adjusted spline curves stratified by the use of aspirin. The pattern of the adjusted HR curve in aspirin users looks different from that in non-users. The adjusted HR of CVE and kidney event appears to increase in patients weighing less than 60 kg in aspirin users, while there seems little difference in risk of CVE and kidney event according to bodyweight in non-users in both unmatched (Supplementary Figures S1 and S2 in supplement) and matched cohort (Fig. 1 and Supplementary Figure S3). The risk of mortality according to bodyweight appears to show the opposite tendency in aspirin users and non-users (Supplementary Figure S1 and Fig. 1), but the numbers of death event (82 out of 2070; 4.0%) were too small to provide a proper answer. Indeed, the analysis failed to reach statistical significance (Table 3). Given the above findings, we evaluated the effects of aspirin on study outcomes in patients weighting less than 60 kg versus those weighting 60 kg or more, separately. In Table 4, the use of aspirin was significantly associated with increased risk of CVE in patients weighing less than 60 kg in both unmatched (HR 2.791; 95% CI 1.218–6.395; *P* = 0.015) and matched cohort (HR 4.014; 95% CI 1.253–12.865, *P* = 0.019). The risk of the composite outcome of CVE or kidney events or death was also significantly increased in aspirin users weighing less than 60 kg in both unmatched and matched cohort. However, in patients weighing 60 kg or more, the use of aspirin was not associated with the risk of any other clinical outcomes.

**Table 3:** Multivariate Cox proportional analyses for clinical outcomes according to the group of bodyweight.

Bw (kg)	CVE		Renal event		All-cause mortality		Composite outcome	
	HR (95% CI)	*P*	HR (95% CI)	*P*	HR (95% CI)	*P*	HR (95% CI)	*P*
**Unmatched cohort (n = 2070)**								
Q1: < 58.5 (n = 515)								
Unadjusted model	2.478 (1.113–5.516)	0.026	1.449 (1.012–2.075)	0.043	1.654 (0.582–4.597)	0.345	1.547 (1.112–2.151)	0.010
Adjusted model	2.776 (1.077–7.153)	0.035	1.292 (0.873–1.912)	0.200	0.840 (0.235–3.000)	0.789	1.331 (0.928–1.908)	0.120
Q2: 58.5–66.0 (n = 506)								
Unadjusted model	2.381 (1.318–4.302)	0.004	1.187 (0.824–1.710)	0.357	1.174 (0.478–2.880)	0.727	1.566 (1.144–2.144)	0.005
Adjusted model	1.247 (0.628–2.478)	0.528	0.911 (0.599–1.386)	0.663	0.531 (0.182–1.546)	0.246	1.005 (0.704–1.434)	0.980
Q3: 66.0–74.0 (n = 520)								
Unadjusted model	1.802 (0.910–3.568)	0.091	1.459 (1.025–2.076)	0.036	1.021 (0.444–2.349)	0.961	1.523 (1.110–2.089)	0.009
Adjusted model	1.325 (0.645–2.722)	0.444	1.068 (0.725–1.573)	0.740	0.419 (0.166–1.060)	0.066	1.037 (0.739–1.455)	0.834
Q4: ≥ 74.0 (n = 529)								
Unadjusted model	2.650 (1.187–5.916)	0.017	1.124 (0.790–1.600)	0.515	1.873 (0.721–4.869)	0.198	1.295 (0.945–1.774)	0.107
Adjusted model	2.438 (0.926–6.419)	0.071	1.374 (0.924–2.043)	0.116	1.544 (0.474–5.032)	0.471	1.370 (0.959–1.957)	0.083
**Matched cohort (n = 1062)**								
Q1: < 60.0 (n = 240)								
Unadjusted model	3.222 (1.171–8.866)	0.024	1.187 (0.793–1.775)	0.405	0.630 (0.206–1.926)	0.417	1.358 (0.933–1.976)	0.110
Adjusted model	4.014 (1.253–12.865)	0.019	1.484 (0.946–2.328)	0.086	0.329 (0.077–1.410)	0.134	1.553 (1.024–2.356)	0.038
Q2: 60.0–67.0 (n = 238)								
Unadjusted model	1.191 (0.581–2.439)	0.634	0.813 (0.525–1.258)	0.352	0.315 (0.112–0.884)	0.028	0.910 (0.621–1.332)	0.626
Adjusted model	1.263 (0.585–2.729)	0.552	0.825 (0.519–1.311)	0.416	0.185 (0.051–0.668)	0.010	0.910 (0.615–1.347)	0.637
Q3: 67.0–75.0 (n = 271)								
Unadjusted model	1.714 (0.623–4.716)	0.297	1.305 (0.832–2.046)	0.246	0.745 (0.308–1.798)	0.512	1.254 (0.843–1.866)	0.264
Adjusted model	1.464 (0.513–4.175)	0.476	1.145 (0.718–1.828)	0.569	1.233 (0.428–3.558)	0.698	1.095 (0.729–1.643)	0.663
Q4: ≥ 75.0 (n = 268)								
Unadjusted model	1.534 (0.595–3.958)	0.376	0.951 (0.605–1.495)	0.827	2.165 (0.417–11.24)	0.358	0.991 (0.661–1.486)	0.966
Adjusted model	2.158 (0.676–6.888)	0.194	1.113 (0.682–1.816)	0.670	2.678 (0.335–21.408)	0.353	1.216 (0.782–1.890)	0.386

Adjusted for age, male gender, BMI, smoking, baseline eGFR, previous CVD, diabetes, hypertension, proteinuria, hemoglobin, albumin and total cholesterol levels, and use of medications (RAAS blockers, CCB, beta-blockers, statin, warfarin, and other antiplatelet agents).

**Table 4:** Multivariate Cox proportional analyses for clinical outcomes according to dichotomized bodyweight (< 60 kg vs. ≥ 60 kg).

	Bodyweight < 60 kg				Bodyweight ≥ 60 kg			
	Unmatched cohort (n = 588)		Matched cohort (n = 240)		Unmatched cohort (n = 1482)		Matched cohort (n = 822)	
	HR (95% CI)	*P*	HR (95% CI)	*P*	HR (95% CI)	*P*	HR (95% CI)	*P*
**CVE**								
Unadjusted model	2.707 (1.357–5.398)	0.005	2.602 (0.928–7.300)	0.069	2.142 (1.429–3.212)	< 0.001	1.498 (0.917–2.446)	0.107
Adjusted model	2.791 (1.218–6.395)	0.015	4.014 (1.253–12.865)	0.019	1.512 (0.978–2.337)	0.063	1.599 (0.968–2.639)	0.067
**Renal event**								
Unadjusted model	1.684 (1.216–2.331)	0.002	1.214 (0.803–1.835)	0.358	1.173 (0.948–1.452)	0.143	0.988 (0.766–1.275)	0.926
Adjusted model	1.377 (0.961–1.972)	0.081	1.484 (0.946–2.328)	0.086	1.070 (0.849–1.348)	0.567	1.017 (0.785–1.318)	0.898
**All-cause mortality**								
Unadjusted model	1.762 (0.669–4.637)	0.251	0.661 (0.210–2.084)	0.480	1.241 (0.739–2.084)	0.414	0.643 (0.357–1.157)	0.141
Adjusted model	0.845 (0.256–2.791)	0.782	0.329 (0.077–1.410)	0.134	0.725 (0.416–1.266)	0.258	0.680 (0.370–1.248)	0.213
**Composite outcome**								
Unadjusted model	1.799 (1.334–2.427)	< 0.001	1.314 (0.892–1.935)	0.167	1.384 (1.147–1.670)	0.001	1.057 (0.845–1.324)	0.626
Adjusted model	1.424 (1.024–1.979)	0.036	1.533 (1.024–2.356)	0.038	1.111 (0.907–1.360)	0.310	1.106 (0.880–1.389)	0.387

Adjusted for age, male gender, BMI, smoking, baseline eGFR, previous CVD, diabetes, hypertension, proteinuria, hemoglobin, albumin and total cholesterol levels, and use of medications (RAAS blockers, CCB, beta-blockers, statin, warfarin, and other antiplatelet agents).

## Discussion

In this study, we found that there was no significant beneficial effect of low-dose aspirin in preventing CVE in patients with CKD. On the contrary, the use of low-dose aspirin was associated with an increased risk of CVE in those patients and this harmful effect was prominent in patients with low bodyweight (< 60 kg). In addition, these adverse effects were insignificant in the primary prevention of CVE, but significant especially in underweight patients in secondary prevention. The risk of all-cause mortality, kidney event, and bleeding was not significantly different between aspirin users and non-users in the CKD cohort.

Low-dose aspirin treatment has shown a beneficial effect in reducing CVE or mortality without increasing significant bleeding risk in patients with the previous CVE, and it is recommended for secondary prevention of CVE^7–9^. However, most of the previous large studies excluded patients with CKD and the beneficial effect of low-dose aspirin in patients with CKD has not been confirmed properly until now.

A meta-analysis reported that antiplatelet therapy in patients with CKD had little or no effect in reducing cardiovascular morbidity and mortality with increased bleeding risk^17^. However, the authors concluded that evidence for antiplatelet therapy in patients with CKD was low or very low quality due to substantial variation and heterogeneity among trials, reliance on subgroup data, and considerable methodological limitations, indicating an uncertainty to the results. A previous study reported that the use of low-dose aspirin in patients with CKD was associated with increased risk for CVD and kidney progression suggesting the harmful effect of aspirin in patients with kidney impairment^18^. Those findings showed similar results in our current study. A recent nationwide study performed in patients with predialysis CKD G5, reported that there was no significant benefit in reducing ischemic stroke, cardiovascular mortality, and all-cause mortality in predialysis advanced CKD patients who received aspirin therapy^19^. In addition, the study showed that the use of aspirin was associated with an increased risk of kidney failure in those patients. These findings also support the negative effect of aspirin use in patients with CKD. Meanwhile, a post-hoc subgroup analysis of HOT (Hypertension Optimal Treatment) study showed that aspirin treatment reduced significantly the risk of CVE and mortality in hypertensive patients with CKD^20^, which was contrary to our findings. However, only 2.9% of participants had an eGFR < 45 mL/min/1.73 m^2^, and a much smaller proportion of participants (0.5%) had an eGFR < 30 mL/min/1.73 m^2^. Moreover, the study was designed for diastolic hypertensive patients and only 8.0% of the study population had diabetes, whereas our study had larger proportion of participants with severe CKD (eGFR < 30 mL/min/1.73 m^2^ in 27.8%) and diabetes (34.0%) than HOT study. These disparities of study population between the two might contribute to different results.

Recently, it was published that low-dose aspirin was effective for the prevention of CVE only in patients with low bodyweight (< 70 kg) and become ineffective with increasing bodyweight^15^. These findings by Rothwell et al. were opposite to our findings in terms of a drug effect (helpful vs. harmful). However, the study by Rothwell et al. and our study showed in common that the effects of aspirin were bodyweight dependent. They suggest that low-dose aspirin therapy is effective in preventing CVE but the efficacy is limited to the people with low bodyweight, while our study suggests that the use of low-dose aspirin in patients with CKD increases the risk for CVE and the patients with low bodyweight are more vulnerable to the harmful effect. These discrepant findings might be attributed to the different study populations between the two studies. They analyzed individual patient data from previous randomized trials that had been conducted to examine the primary and secondary preventive effects of aspirin. The subjects of this study, which consisted only of patients with CKD, were different from the previous one, and it can be assumed that the intensity of the aspirin effect according to the bodyweight due to race differences might lead to different results.

In this study, the use of low-dose aspirin in patients with CKD was shown to may be associated with an increased risk of CVE. A possible explanation for the discrepancies in the effects of low-dose aspirin between the CKD and non-CKD population is that kidney function affects the efficacy of low-dose aspirin. A Japanese demonstrated that low-dose aspirin therapy reduced the incidence of CVE in patients with estimated glomerular filtration rate based on serum creatinine (eGFR_cr_) 60–90 mL/min/1.73 m^2^, but not in patients with eGFR_cr_ < 60 mL/min/1.73 m^2^ or eGFR_cr_ ≥ 90 mL/min/1.73 m^2^ ^21^, and suggested that eGFR_cr_ might affect the efficacy of low-dose aspirin therapy in those patients.

Insufficient antiplatelet effect referred to as the high on-treatment platelet reactivity (HTPR) is known to be associated with an increased incidence of CVE and mortality in patients taking antiplatelet medications for secondary prevention^22,23^. It has been shown that HTPR is more frequent in patients with CKD than in those with normal kidney function^24,25^. It means that impaired kidney function attenuates the effects of antiplatelet agents. Another potential explanation for our finding is that patients with CKD have non-traditional CKD-related risk factors for CVD, which are not influenced by aspirin therapy. The non-traditional risk factors including uremia, chronic inflammation, mineral bone metabolism, and oxidative stress are known to be associated with increased CVD morbidity and mortality in patients with CKD^26,27^. Aspirin paradox may be a plausible mechanism supporting our hypothesis. Aspirin exerts anti-thrombotic effects by the inhibition of cyclooxygenase-1 (COX-1) that leads to a decrease in the production of thromboxane A_2_, while aspirin also exerts pro-thrombotic effects by the inhibition of COX-2 that induces a decrease in the production of prostacyclin (PGI_2_). Usually, low-dose aspirin exhibits higher affinity for COX-1, while high-dose aspirin inhibits both COX-1 and COX-2^28^. However, there were several studies suggesting that low-dose aspirin could inhibit COX-2 and induce pro-thrombotic effects. In some experiments, it was shown that low-dose aspirin inhibits COX-2 leading to pro-thrombotic effects^29,30^. Actually, a recent study of patients with diabetes reported that the use of low-dose aspirin was associated with an increased risk of ischemic stroke^31^, suggesting that low-dose aspirin could induce pro-thrombotic effects. These findings also support our hypothesis. However, the above-mentioned speculations are all merely hypothetical for the observed findings. To explain the underlying mechanism, further well-organized and sophisticated experimental and clinical studies are needed.

Although we drew the conclusion using a large-scale prospective CKD cohort study that included patients with CKD across all stages of the disease, there are some limitations to be discussed. First, this study was an observational study, not a randomized trial. Thus, it had inherently flaw in the randomized allocation of participants to the treatment (users of aspirin) or the control arm (non-users of aspirin), which could result in selection bias. Confounding by indication for aspirin use may exert an influence on the outcome. To minimize such selection bias and confounding, we used the PS matching method and confirmed that baseline covariates have been balanced between the two groups. However, this is not a complete method to substitute for the randomized trial, and unrecognized bias can remain and affect our conclusion. Second, this study was conducted only in one ethnic people. One of our findings was that the harmful effect of low-dose aspirin in patients with CKD was bodyweight-dependent. There have been accumulating data about ethnic or racial differences in bodyweight and disease risk^32–34^. In addition, aspirin intolerance varies by race and ethnicity and the racial and ethnic variations are known to be related to genetic polymorphic metabolism^35,36^. There have been several reports for a different efficacy of aspirin by race and ethnicity which were studied in patients with cancers^37,38^ or preeclampsia^39^. Considering these data, the effect of low-dose aspirin might be different by race and ethnicity in CKD patients. Third, our analysis did not reflect the individual’s bodyweight change during the follow-up. Fourth, we cannot know whether discontinuing or not-using low-dose aspirin in patients with CKD improve cardiovascular outcomes because this was not an interventional study. Fifth, non-traditional CKD-related risk factors for CVD such as inflammation and disordered mineral bone metabolism might confound the effect of aspirin on outcome. However, we did not have sufficient data on those markers and could not exclude the confounding by inflammation or mineral metabolism.

In conclusion, this study showed that the use of low-dose aspirin in patients with CKD was associated with a significant increase of CVE without improving all-cause mortality and this harmful effect of low-dose aspirin was prominent in patients with low bodyweight (< 60 kg). Therefore, our results do not support prescribing low-dose aspirin routinely for the prevention of CVE in patients with CKD, particularly patients with low bodyweight. Further studies are warranted to verify our results and determine the mechanism underlying our findings.

## Methods

### Study design and participants

The KoreaN cohort study for Outcome in patients With Chronic Kidney Disease (KNOW-CKD) is a prospective, nationwide, multicenter, and observational cohort study. In brief, 2238 adults aged 20 to 75 years with CKD G1-5 (non-dialysis) were enrolled between 2011 and 2016 (NCT01630486 at http://www.clinicaltrials.gov) from nine tertiary hospitals in Korea. The study design, method, and protocol summary are described in detail elsewhere^40^. For this study, we excluded participants who did not have anthropometric measurements at baseline (n = 24) and other baseline covariates (n = 144). Therefore, a total of 2070 participants were included in the final analysis.

This study was conducted in accordance with the Declaration of Helsinki, and the research protocol was approved by the institutional review boards of the Seoul National University Hospital (1104–089–359), Seoul National University Bundang Hospital (B-1106/129-008), Yonsei University Severance Hospital (4-2011-0163), Kangbuk Samsung Medical Center (2011-01-076), Seoul St. Mary’s Hospital (KC11OIMI0441), Gachon University Gil Medical Center (GIRBA2553), Eulji General Hospital (201105-01), Chonnam National University Hospital (CNUH-2011-092), and Pusan Paik Hospital (11–091). Written informed consent was obtained from all subjects.

### Data collection and measurements

Socio-demographic data including age, sex, smoking history, medication, and personal and family medical history were recorded at enrollment. These baseline data were collected from self-reported questionnaire and review of medical records. Anthropometric measurements such as height, weight, and body mass index (BMI) were collected at baseline. For medical history, CVD was defined as any history of coronary artery disease, congestive heart failure, cerebrovascular disease, and peripheral artery disease. The eGFR_cr_ was calculated using the Chronic Kidney Disease Epidemiology Collaboration (CKD-EPI) equation with serum creatinine^41^. Spot urine protein-creatinine ratio (uPCR) was used for the assessment of proteinuria. Patients were regularly followed up according to the study protocol, and the events related to study outcomes were recorded during the follow-ups. Patients were censored at the last follow up if they were lost to follow up. Death was investigated by reviewing medical records or using data from the National Database of Statistics Korea. The patients who were lost to follow up have been traced for the information on survival and cause of death with the help of the National Health Insurance System and Korea Statistical Information Service.

### Outcomes

The primary outcome was the first occurrence of major CVE which includes acute myocardial infarction, unstable angina, receiving percutaneous coronary artery intervention (PCI) or coronary bypass graft surgery (CABG), stroke, cerebral hemorrhage, congestive heart failure, and other CVE that required hospitalization or interventional treatment. The secondary outcomes were kidney events defined as a > 50% reduction of eGFR_cr_ from baseline, doubling of serum creatinine, or onset of kidney failure with replacement therapy, all-cause mortality, the composite outcome of the CVE or kidney event or death, and bleeding event.

### Statistical analyses

The data were presented as frequencies and percentages for categorical variables and as mean ± SD for continuous variables. PS matching was used to reduce the selection bias due to the lack of random assignment. To predict the probability of aspirin treatment, a multiple logistic regression was constructed using the following covariates; age, sex, BMI, smoking history, diabetes, hypertension, CVD, eGFR_cr_, uPCR, serum levels of hemoglobin, albumin, total cholesterol, and the use of renin–angiotensin–aldosterone system (RAAS) blockers, calcium channel blockers (CCBs), beta-blockers, and statins. After calculating the PS of participants, we subsequently matched aspirin users to non-users using PS based on near-neighbor with calipers method^42,43^. To assess the effect of aspirin use on the incidence of outcomes, we performed multivariable Cox proportional hazards models and estimated hazard ration (HR) and 95% confidence intervals (CIs) of aspirin use. The multivariable Cox model was adjusted for age, male gender, BMI, smoking, baseline eGFR, previous CVD, diabetes, hypertension, proteinuria, hemoglobin, albumin and total cholesterol levels, and use of medications (RAAS blockers, CCB, beta-blockers, statin, warfarin, and other antiplatelet agents). We explored the incidence of study outcomes according to bodyweight in the stratified analysis by aspirin use by plotting an adjusted spline curve for HR of outcomes. All statistical analyses were performed using R software, version 3.5.3 with packages (The Comprehensive R Archive Network: http://cran.r-project.org).

### Patient and public involvement

Patients and/or the public were not involved in the design, or conduct, or reporting, or dissemination plans of this research.

## Supplementary Information

Supplementary Informations.

## Data Availability

Data are available on reasonable request. The corresponding author has full access to all data in the study and final responsibility for the submission of the article for publication. Due to data security reasons (ie, data contain potentially participant identifying information), the KNOW-CKD study does not allow sharing data as a public use file. Data requests can also be addressed to: jyjung@gachon.ac.kr.

## References

[1] Coresh J (2007). Prevalence of chronic kidney disease in the United States. JAMA.

[2] Smith DH, Gullion CM, Nichols G, Keith DS, Brown JB (2004). Cost of medical care for chronic kidney disease and comorbidity among enrollees in a large HMO population. J. Am. Soc. Nephrol..

[3] Hunsicker LG (2004). The consequences and costs of chronic kidney disease before ESRD. J. Am. Soc. Nephrol..

[4] Go AS, Chertow GM, Fan D, McCulloch CE, Hsu CY (2004). Chronic kidney disease and the risks of death, cardiovascular events, and hospitalization. N. Engl. J. Med..

[5] de Jager DJ (2009). Cardiovascular and noncardiovascular mortality among patients starting dialysis. JAMA.

[6] Mathew RO (2017). Diagnosis and management of atherosclerotic cardiovascular disease in chronic kidney disease: a review. Kidney Int..

[7] Antithrombotic Trialists C (2002). Collaborative meta-analysis of randomised trials of antiplatelet therapy for prevention of death, myocardial infarction, and stroke in high risk patients. BMJ.

[8] Antiplatelet Trialists' Collaboration (1994). Collaborative overview of randomised trials of antiplatelet therapy—I: prevention of death, myocardial infarction, and stroke by prolonged antiplatelet therapy in various categories of patients. BMJ.

[9] Baigent C (2009). Aspirin in the primary and secondary prevention of vascular disease: collaborative meta-analysis of individual participant data from randomised trials. Lancet.

[10] Ong G, Davis TM, Davis WA (2010). Aspirin is associated with reduced cardiovascular and all-cause mortality in type 2 diabetes in a primary prevention setting: the Fremantle Diabetes study. Diabetes Care.

[11] ETDRS Investigators (1992). Aspirin effects on mortality and morbidity in patients with diabetes mellitus: early treatment diabetic retinopathy study report 14. JAMA.

[12] Ogawa H (2008). Low-dose aspirin for primary prevention of atherosclerotic events in patients with type 2 diabetes: a randomized controlled trial. JAMA.

[13] Belch J (2008). The prevention of progression of arterial disease and diabetes (POPADAD) trial: factorial randomised placebo controlled trial of aspirin and antioxidants in patients with diabetes and asymptomatic peripheral arterial disease. BMJ.

[14] Sasso FC (2015). Lack of effect of aspirin in primary CV prevention in type 2 diabetic patients with nephropathy: results from 8 years follow-up of NID-2 study. Acta Diabetol..

[15] Rothwell PM (2018). Effects of aspirin on risks of vascular events and cancer according to bodyweight and dose: analysis of individual patient data from randomised trials. Lancet.

[16] Dreisbach AW, Lertora JJL (2008). The effect of chronic renal failure on drug metabolism and transport. Expert Opin. Drug Metab. Toxicol..

[17] Palmer SC (2012). Effects of antiplatelet therapy on mortality and cardiovascular and bleeding outcomes in persons with chronic kidney disease: a systematic review and meta-analysis. Ann. Intern. Med..

[18] Kim AJ (2014). Low-dose aspirin for prevention of cardiovascular disease in patients with chronic kidney disease. PLoS ONE.

[19] Hsiao KC (2017). Different impact of aspirin on renal progression in patients with predialysis advanced chronic kidney disease with or without previous stroke. Eur. J. Intern. Med..

[20] Jardine MJ (2010). Aspirin is beneficial in hypertensive patients with chronic kidney disease: a post-hoc subgroup analysis of a randomized controlled trial. J. Am. Coll. Cardiol..

[21] Saito Y (2011). Low-dose aspirin therapy in patients with type 2 diabetes and reduced glomerular filtration rate: subanalysis from the JPAD trial. Diabetes Care.

[22] Gum PA, Kottke-Marchant K, Welsh PA, White J, Topol EJ (2003). A prospective, blinded determination of the natural history of aspirin resistance among stable patients with cardiovascular disease. J. Am. Coll. Cardiol..

[23] Mayer K (2014). Aspirin treatment and outcomes after percutaneous coronary intervention: results of the ISAR-ASPI registry. J. Am. Coll. Cardiol..

[24] Angiolillo DJ (2010). Impact of chronic kidney disease on platelet function profiles in diabetes mellitus patients with coronary artery disease taking dual antiplatelet therapy. J. Am. Coll. Cardiol..

[25] Gremmel T (2013). Chronic kidney disease is associated with increased platelet activation and poor response to antiplatelet therapy. Nephrol. Dial. Transplant..

[26] Vlagopoulos PT, Sarnak MJ (2005). Traditional and nontraditional cardiovascular risk factors in chronic kidney disease. Med. Clin. North Am..

[27] Weiner DE (2008). The relationship between nontraditional risk factors and outcomes in individuals with stage 3 to 4 CKD. Am. J. Kidney Dis..

[28] Majed BH, Khalil RA (2012). Molecular mechanisms regulating the vascular prostacyclin pathways and their adaptation during pregnancy and in the newborn. Pharmacol. Rev..

[29] Jaffe EA, Weksler BB (1979). Recovery of endothelial cell prostacyclin production after inhibition by low doses of aspirin. J. Clin. Investig..

[30] Doutremepuich C, Aguejouf O, Eizayaga FX, Desplat V (2007). Reverse effect of aspirin: is the prothrombotic effect after aspirin discontinuation mediated by cyclooxygenase 2 inhibition?. Pathophysiol. Haemost. Thromb..

[31] Kim YJ (2015). Evaluation of low-dose aspirin for primary prevention of ischemic stroke among patients with diabetes: a retrospective cohort study. Diabetol. Metab. Syndr..

[32] Wen CP (2009). Are Asians at greater mortality risks for being overweight than Caucasians? Redefining obesity for Asians. Public Health Nutr..

[33] Pan WH (2004). Body mass index and obesity-related metabolic disorders in Taiwanese and US whites and blacks: implications for definitions of overweight and obesity for Asians. Am. J. Clin. Nutr..

[34] Consultation WHOE (2004). Appropriate body-mass index for Asian populations and its implications for policy and intervention strategies. Lancet.

[35] Jose AGA (2009). Pharmacogenomics in aspirin intolerance. Curr. Drug Metab..

[36] FitzGerald R, Pirmohamed M (2011). Aspirin resistance: effect of clinical, biochemical and genetic factors. Pharmacol. Ther..

[37] Smith CJ (2017). Aspirin use reduces the risk of aggressive prostate cancer and disease recurrence in African–American men. Cancer Epidemiol. Biomark. Prev..

[38] Erickson P (2018). Racial and ethnic differences in the relationship between aspirin use and non-small cell lung cancer risk and survival. Cancer Epidemiol. Biomark. Prev..

[39] Tolcher MC, Sangi-Haghpeykar H, Mendez-Figueroa H, Aagaard KM (2020). Low-dose aspirin for preeclampsia prevention: efficacy by ethnicity and race. Am. J. Obstet. Gynecol. MFM.

[40] Oh KH (2014). KNOW-CKD (KoreaN cohort study for Outcome in patients With Chronic Kidney Disease): design and methods. BMC Nephrol..

[41] Levey AS (2009). A new equation to estimate glomerular filtration rate. Ann. Intern. Med..

[42] Ho D, Imai K, King G, Stuart EA (2011). MatchIt: nonparametric preprocessing for parametric causal inference. J. Stat. Softw..

[43] Olmos A, Govindasamy P (2015). Propensity scores: a practical introduction using R. J. MultiDiscip. Eval..

