# Supplementary material for: Low-dose aspirin was associated with an increased risk of cardiovascular events in patients with chronic kidney disease and low bodyweight: results from KNOW-CKD study
Source: Sci Rep. 2021 Mar 23;11:6691. doi: 10.1038/s41598-021-86192-4 (PMC7988000; doi:10.1038/s41598-021-86192-4)
Supplement: Supplementary file 1 — Supplementary Informations. [file 41598_2021_86192_MOESM1_ESM.docx]

**Supplemental Material**

**Low-dose aspirin was associated with an increased risk of cardiovascular events in patients with chronic kidney disease patients and low bodyweight: Results from KNOW-CKD study**

Yun Jung Oh, MD, PhD ^1,2^; Ae Jin Kim, MD, PhD^3,4^; Han Ro, MD, PhD^3,4^; Jae Hyun Chang, MD, PhD^3,4^; Hyun Hee Lee, MD, PhD^3,4^; Wookyung Chung, MD, PhD^3,4^; Young Youl Hyun, MD, PhD^5^; Joongyub Lee, MD, PhD^6^; Yeong Hoon Kim, MD, PhD^7^; Seung Hyeok Han, MD, PhD^8^; Dong-Wan Chae, MD, PhD^9^; Curie Ahn, MD, PhD^10^; Kook-Hwan Oh, MD, PhD^10^; and Ji Yong Jung, MD, PhD^1,3,4^

^1^Department of Internal Medicine, Graduate School of Medicine, Gachon University, Incheon, Republic of Korea; ^2^Division of Nephrology, Department of Internal Medicine, Cheju Halla General Hospital, Cheju, Republic of Korea; ^3^Division of Nephrology, Department of Internal Medicine, Gil Medical Center, Incheon, Republic of Korea; ^4^College of Medicine, Gachon University, Incheon, Republic of Korea; ^5^Department of Internal Medicine, Sungkyunkwan University School of Medicine, Kangbuk Samsung Hospital, Seoul, Republic of Korea; ^6^Department of Prevention and Management, School of Medicine, Inha University, Incheon, Republic of Korea; ^7^Department of Internal Medicine, Busan Paik Hospital, College of Medicine, Inje University, Busan, Republic of Korea; ^8^Department of Internal Medicine, College of Medicine, Institute of Kidney Disease Research, Yonsei University, Seoul, Republic of Korea; ^9^Department of Internal Medicine, Seoul National University Bundang Hospital, Seoul, Republic of Korea; ^10^Department of Internal Medicine, Seoul National University Hospital, Seoul, Republic of Korea

**Running Title:** Low dose Aspirin in CKD

To whom correspondence should be addressed:

**Dr. Ji Yong Jung,** Division of Nephrology, Department of Internal Medicine, Gachon University Gil Medical Center, Gachon University College of Medicine, 21, Namdong-daero 774 beon-gil, Namdong-gu, Incheon, 21565, Republic of Korea. Tel: +82 32 458 2621; Fax: +82 32 460 3431; E-mail: jyjung@gachon.ac.kr

**Table S1. Adjusted HR of aspirin use for CVE according to eGFR**

|  | eGFR ≥60 ml/min/1.73m^2^ | | | | |  | eGFR <60 ml/min/1.73m^2^ | | | | |
| --- | --- | --- | --- | --- | --- | --- | --- | --- | --- | --- | --- |
|  | Unmatched cohort (n=728) | |  | Matched cohort (n=258) | |  | Unmatched cohort (n=1,342) | |  | Matched cohort (n=804) | |
|  | HR (95% CI) | *P* |  | HR (95% CI) | *P* |  | HR (95% CI) | *P* |  | HR (95% CI) | *P* |
| Overall | 1.200 (0.536-2.688) | 0.658 |  | 0.725 (0.282-1.861) | 0.504 |  | 1.748 (1.127-2.713) | 0.013 |  | 2.143 (1.258-3.650) | 0.005 |
| Bodyweight < 60kg | 0.599 (0.109-3.283) | 0.555 |  | 2.825e+07 (0-Inf) | 0.990 |  | 4.612 (1.600-13.294) | 0.005 |  | 4.410 (1.097-17.754) | 0.037 |
| Bodyweight ≥ 60kg | 1.190 (0.418-3.388) | 0.745 |  | 0.743 (0.218-2.540) | 0.636 |  | 1.435 (0.880-2.341) | 0.147 |  | 1.839 (1.022-3.308) | 0.042 |

Adjusted for age, male gender, BMI, smoking, baseline eGFR, previous CVD, diabetes, hypertension, proteinuria, hemoglobin, albumin and total cholesterol levels, and use of medications (RAAS blockers, CCB, beta-blockers, statin, warfarin, and other antiplatelet agents).

**
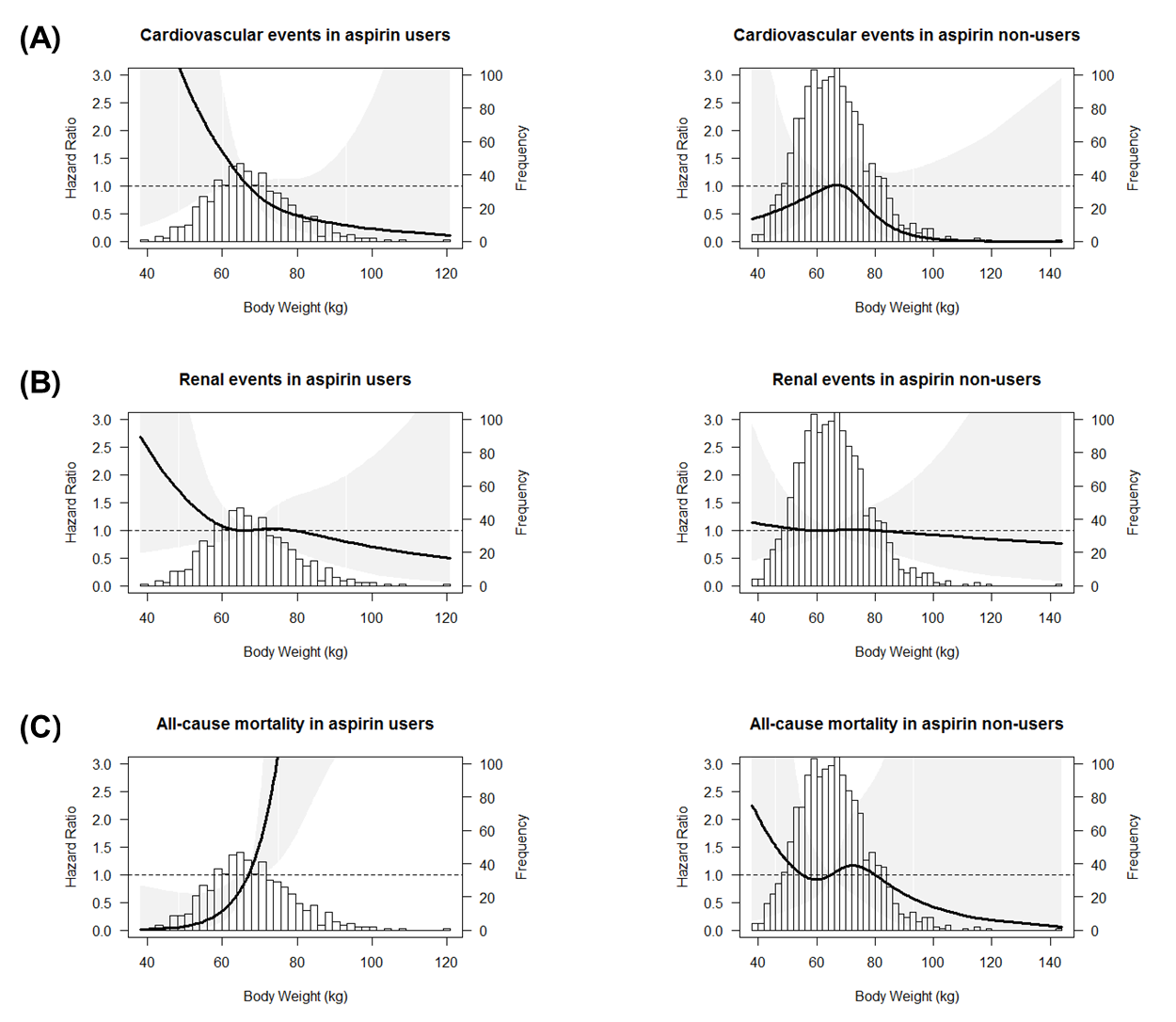
 Supplemental Figure S1. Adjusted risk of clinical outcomes according to bodyweight in aspirin users versus non-users before PS matching.** The spine curves show the adjusted hazard ratio of (A) cardiovascular events, (B) renal events, and (C) all-cause mortality in aspirin users versus non-users. Hazard ratios were adjusted for use of age, sex, BMI, smoking history, diabetes, hypertension, CVD, eGFR_cr_, uPCR, serum levels of hemoglobin, albumin, total cholesterol, and the use of RAAS blockers, CCBs, beta-blockers, statins, warfarin, and other antiplatelet. The histograms represent the frequency of distribution of bodyweight.

**
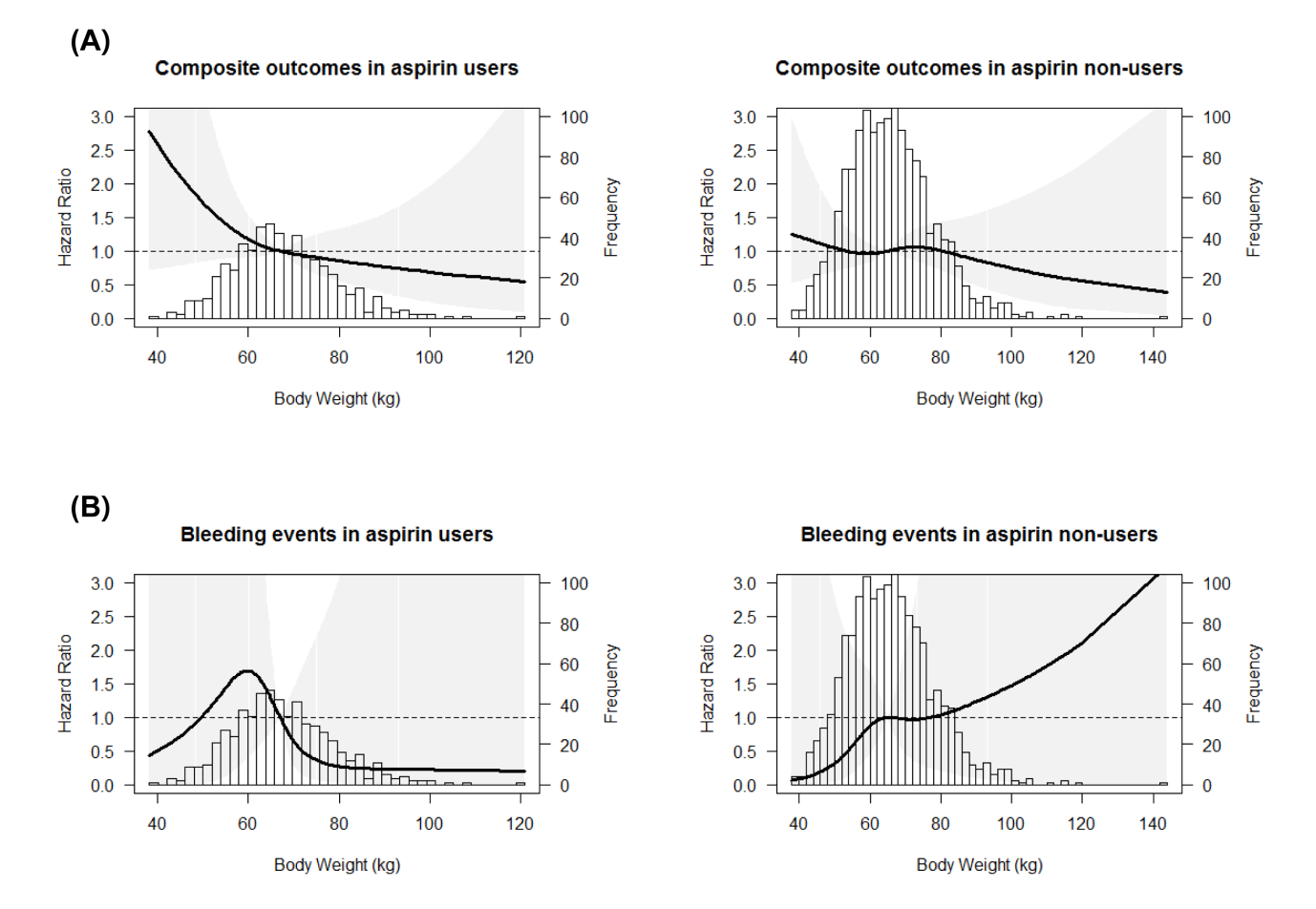
**

**Supplemental Figure S2. Adjusted risk of clinical outcomes according to body weight in aspirin users versus non-users before PS matching.** The spine curves show the adjusted hazard ratio of of (A) the composite outcome of the CVE or renal event or death, and bleeding event, (B) bleeding events. Hazard ratios were adjusted for use of age, sex, BMI, smoking history, diabetes, hypertension, CVD, eGFR_cr_, uPCR, serum levels of hemoglobin, albumin, total cholesterol, and the use of RAAS blockers, CCBs, beta-blockers, statins, warfarin, and other antiplatelet agents. The histograms represent the frequency of distribution of body weight.

**
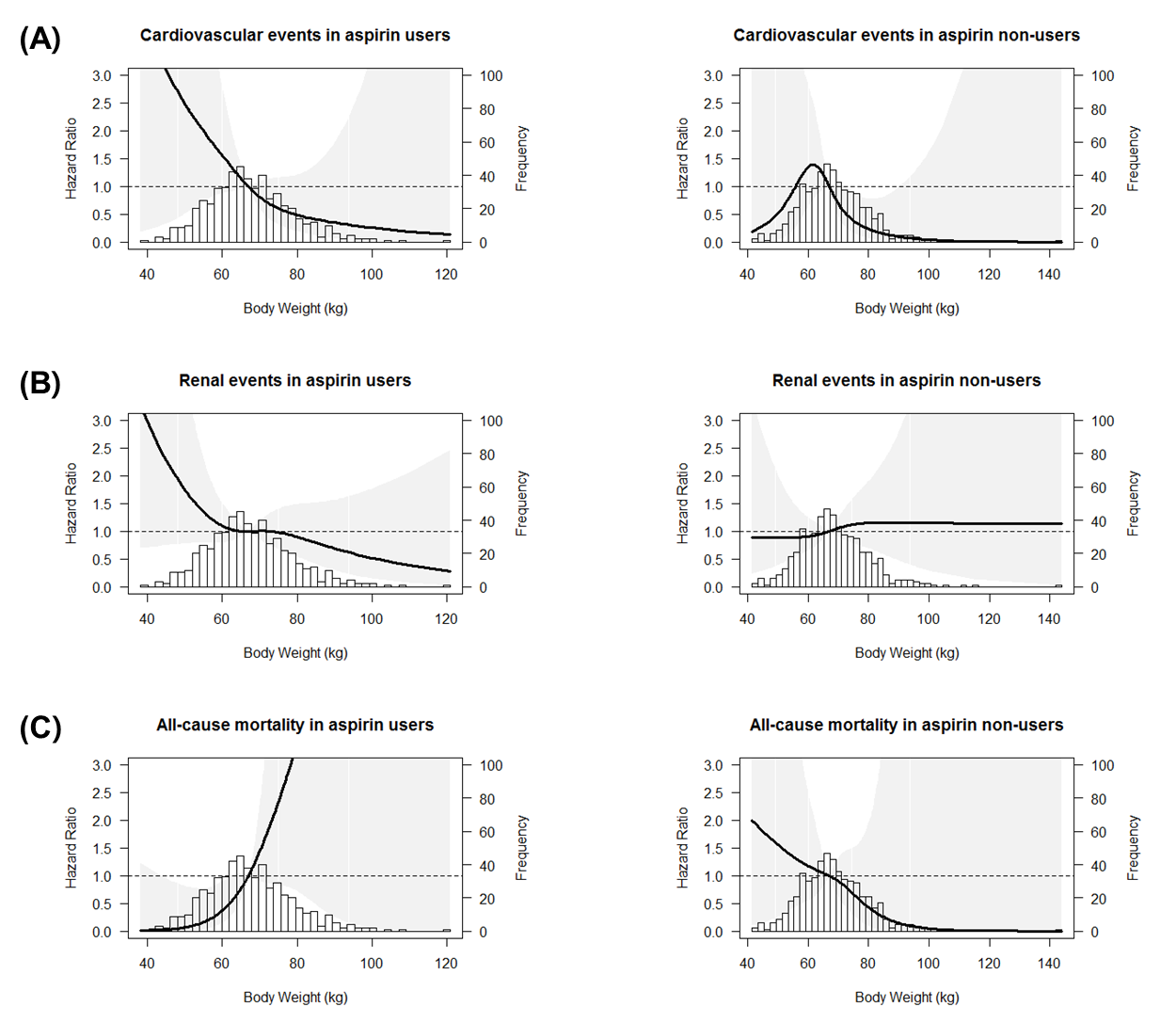
**

**Supplemental Figure S3. Adjusted risk of clinical outcomes according to bodyweight in aspirin users versus non-users after PS matching.** The spine curves show the adjusted hazard ratio of (A) cardiovascular events, (B) renal events, and (C) all-cause mortality in aspirin users versus non-users. Hazard ratios were adjusted for use of age, sex, BMI, smoking history, diabetes, hypertension, CVD, eGFR_cr_, uPCR, serum levels of hemoglobin, albumin, total cholesterol, and the use of RAAS blockers, CCBs, beta-blockers, statins, warfarin, and other antiplatelet. The histograms represent the frequency of distribution of bodyweight.
